# Validation and Translation of the Relational Aspect of Care Questionnaire into the Malay Language (RAC-QM) to Evaluate the Compassionate Care Level of Healthcare Workers from the Patient’s Perspective

**DOI:** 10.3390/ijerph192013486

**Published:** 2022-10-18

**Authors:** Noorhidayu Monyati Mohamed Noor, Mohd Ismail Ibrahim, Suhaily Mohd Hairon, Maizun Mohd Zain, Mohd Saiful Nazri Satiman

**Affiliations:** 1 Department of Community Medicine, School of Medical Sciences, Universiti Sains Malaysia, Kubang Kerian, Kota Bharu 16150, Kelantan, Malaysia; 2 Public Health Unit, Hospital Raja Perempuan Zainab II, Kota Bharu 16150, Kelantan, Malaysia; 3 Medical Division, Kelantan State Health Department, Kota Bharu 16150, Kelantan, Malaysia

**Keywords:** compassionate care, relational aspect of care questionnaire, confirmatory factor analysis, validity, reliability

## Abstract

Background: Compassionate care has been increasingly highlighted in the past few decades worldwide, including in Malaysia. Despite acknowledging its importance, Malaysia still lacks a validated tool that can be used to assess the level of compassionate care from the patient’s perspective. Therefore, this study aims to validate and translate the Relational Aspect of Care Questionnaire (RAC-Q) into the Malay language. Methods: Permission to use and translate the original RAC-Q into the Malay language was obtained. The RAC-Q was then translated into the Malay language following the 10 steps proposed for the translation of a patient-reported outcome questionnaire. A pretest was conducted based on 30 inpatients to assess the appropriateness and clarity of the finalized translated questionnaire. A cross-sectional study was performed based on 138 inpatients from six adult wards of a teaching hospital so as to validate the translated questionnaire. The data were analyzed using R software version 4.1.3 (R Core Team, Vienna, Austria, 2020). The results were presented descriptively as numbers and percentages or means and standard deviations. A confirmatory factor analysis was performed using robust estimators. Results: The analysis showed that the measurement model of the RAC-Q Malay version (RAC-QM) fits well based on several fit indices: a standardized factor loading range from 0.40 to 0.73, comparative fit index (CFI) of 0.917, Tucker–Lewis fit index (TLI) of 0.904, root mean square error of approximation (RMSEA) of 0.06, and a standardized root mean square residual (SRMR) of 0.073. It has good reliability, with a Cronbach’s alpha of 0.857 and a composite ratio of 0.857. Conclusion: The RAC-QM demonstrated good psychometric properties and is valid and reliable based on the confirmatory analysis, and it can thus be used as a tool for evaluating the level of compassionate care in Malaysia.

## 1. Introduction

The evaluation of patients’ experience when receiving healthcare services has become an important topic worldwide, especially in the past few decades. In 2001, the Institute of Medicine (IOM) released its Patient Safety Goals, which emphasized patient-centered care as one of the goals [1]. Before then, little attention was given to this aspect of quality care. This could be attributed to many factors, such as the shifting of tax-funded healthcare systems to privatized and performance-based payments. The revolution of industries also places constant pressure on healthcare systems worldwide to take into account patients’ experiences of receiving care. In general, a good patient experience should encompass all aspects of care, which include the functional, transactional, and relational aspects of care. The scarcity of resources in the healthcare system should not act as a barrier to the provision of high-quality care, which includes all aspects of their management. It is certain that a good patient experience not only improves the well-being of patients but also benefits the healthcare system as a whole. However, these aspects of care are, indeed, very complex and depend on the subjective experiences of individual patients [2], especially the relational aspect. Much work has been conducted in order to develop tools that can help us to accurately measure the relational aspect of care so that improvements can be made. Most of them have been developed for a unique target population.

The importance of the relational aspect of care has also been emphasized in many other reports [3,4,5]. One of the infamous public inquiry reports that highlighted compassionate care is the Francis Report [6]. The inquiry was conducted as a consequence of many public complaints against the Mid-Staffordshire National Health Service (NHS) Foundation Trust concerning poor care. The report revealed many weaknesses in the system, but the most prominent was the lack of compassion among its staff. The report also highlighted the need for a measurement tool with which to properly assess compassion [6]. The Picker Institute Europe is among the organizations dedicated to nurturing compassionate care in the healthcare system. The organization has developed many measurement tools to cater to different target groups or healthcare settings. One of them is the Relational Aspect of Care Questionnaire (RAC-Q), which has been widely used across the UK. Many other measurement tools are being developed, validated, and reviewed. Nevertheless, a review of nine studies that reported on the measurement tools for compassionate care in healthcare found that there is still an unmet need for a psychometrically validated tool that can comprehensively measure the construct of compassion in healthcare settings [7]. 

**Figure 1:**
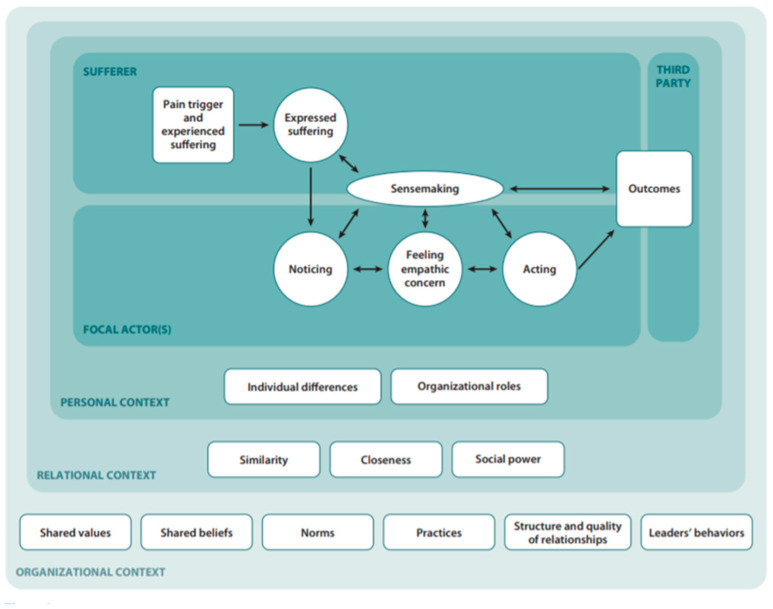
Model of the Interpersonal Process of Compassion.

In Malaysia, efforts to improve patient experiences can be seen in many initiatives. Among the earliest was the incorporation of *budaya penyayang* or “caring” as one of the central values of the Ministry of Health’s (MOH) corporate culture. MOH’s corporate culture committee was formed in the 1990s, with three central values: caring, professionalism, and teamwork. The committee undertook many initiatives in order to instill these values through corporate songs, workshops, and exhibitions. However, after more than three decades since its introduction, there was very limited published evidence regarding the program’s effectiveness, especially from the patient’s perspective. MOH received over five thousand official complaints each year, and at least a quarter of the complaints were related to poor service quality [8]. However, contrary to the common belief that the lack of compassionate care is due to healthcare worker’s (HCW) weakness and ignorance, evidence showed that patients’ characteristics and the care environment considerably affect patients’ perspectives on the compassionate care provided [9,10,11,12]. As long as these factors are not recognized, the quality of our image of healthcare will be jeopardized. The Model of the Interpersonal Process of Compassion [9], as shown in Figure 1, highlights the complexity of compassionate care, which involves many aspects related to different parties. The lack of a clinical measure of compassion with solid evidence of the measurements’ validity is a significant barrier to the improvement and development of clinical practice and patient satisfaction [10]. Therefore, a psychometrically valid measurement tool is needed in order to correctly measure this crucial aspect of care.

To date, the RAC-Q is among the available, validated tools used to measure healthcare workers’ compassionate care for inpatients. The RAC-Q was developed and validated by researchers from the Picker Institute Europe and Nuffield Department of Population Health, University of Oxford. It is based on data from the 2012 NHS Emergency Department Survey and the 2013 NHS Adult Inpatient Survey. The initial questionnaire contained 20 closed-ended questions, with a very high reliability (factor loading of 0.458–0.870, Cronbach’s alpha of 0.950, and McDonald’s omega of 0.951). It was then subsequently reduced to a 12-item questionnaire. The short version of the questionnaire has a better completion rate but is still able to retain its strong psychometric properties (Cronbach’s alpha of 0.92 and intraclass correlation coefficient of 0.97). It measures the relational aspect of care across 22 themes [11]. The completion of the survey requires a mean time of 8.5 min, with a standard deviation of 9.9 min [12]. The original RAC-Q was administered digitally, and the responses were captured in near real time. Therefore, prompt actions could be taken by the service provider in response to the feedback received. A similar method can be applied to assess and improve compassionate care among healthcare workers in Malaysia. However, for this purpose, the RAC-Q would need to be translated, since most respondents will be Malays. Linguistic appropriateness is one of the key factors that can improve the results’ validity and reliability [13,14].

## 2. Materials and Methods

### 2.1. Study Setting and Participants

Owing to the fact that the data collection was carried out during the COVID-19 pandemic, along with the Movement Control Order (MCO) in Malaysia, several special considerations had to be taken. A teaching hospital in Kelantan, Malaysia, was selected as the study location. A prior meeting with the head nurse was conducted to obtain an overview of the wards’ usage, since many wards had been reassigned for COVID-19 cases during the pandemic. There were also strict measures regarding access to the wards. After a thorough discussion of the study, it was concluded that there were only six adult wards suitable for the study, namely, the surgical ward, orthopedic ward, medical ward, and oncology ward, as well as the obstetrics and gynecological ward and a multidisciplinary executive ward. According to Kline, for freely estimated parameters, a minimum of 10 samples used to represent each item can provide adequate statistical power [14]. The RAC-Q contains 12 items; therefore, a minimum of 120 samples were required. A high dropout rate of 20% was applied because of unpredictable bed occupancy rates and the capacity of the inpatients to answer the questionnaire during the pandemic.

A second briefing session with the head nurses from each ward was held. Details of the study, including how it would be conducted, its purpose, and its benefits, were explained. The standard operating procedures, in accordance with guidelines for conducting research during the MCO, were carefully discussed. The expected daily bed occupancy rate was also noted during the meeting to help us in determining the sampling size. A stratified sampling proportionate to the number of beds was conducted in order to determine the number of participants needed from each ward. The executive ward had the lowest number of beds, followed by the oncology ward. The medical, surgical, and orthopedic wards had similar numbers of beds, whereas the obstetrics and gynecology ward had double the number of beds compared with the rest. A minimum of five days and a maximum of ten days were allocated to each ward in order to complete the data collection. On the day of the data collection, the eligible study population was determined according to strict criteria. Study participants were selected through computer-generated random numbers based on their bed numbers. The patients selected for the study were those aged 18 and above who had been in the ward for at least 24 h, were able to read and write, and were clinically stable enough to answer the questionnaire by themselves. Those who consented to participate were given the paper-based questionnaire and were asked to read the patient information sheet and answer the questions by themselves. They were also encouraged to watch a short video about the study, which was provided through a QR-coded link. Completed questionnaires were collected afterward by the researcher. 

### 2.2. Relational Aspect of Care Questionnaire (RAC-Q)

The RAC-Q was developed in 2017 in response to the Francis Report that highlighted the lack of compassionate care as the crucial cause that led to the failure of the UK Mid-Staffordshire NHS Foundation Trust. One of the report’s important recommendations was the need for a reliable measurement tool that could be sued to correctly measure compassionate care. The Picker Institute Europe, a non-governmental organization dedicated to compassionate care, collaborated with the University of Oxford to develop a questionnaire measuring this critical aspect of relational care, namely, compassionate care. The questionnaire was developed using a combination of quantitative, qualitative, and participatory research approaches [11]. During the initial development process, they identified 22 themes that included patient-perceived levels of security, knowledge, and personal value during their hospital stay or visit. The original questionnaire contained 20 items but was then successfully reduced to 12 items with only one domain. Five of the questions had a three-point Likert scale, whereas the other seven questions used four points. The responses were either in agreement form or in frequency form. For the answers in agreement form, four points were given for “definitely true”, three points were given for “mostly true”, one point was given for “definitely false”, and two points were given for “unsure”. For the answers in frequency form, four points were given for “every time”, three points were given for “sometimes,” one point was given for “never,” and two points were given for “unsure”. The total score given by the patients was then converted into percent. The survey tool kit was widely used in regions under the UK’s NHS. However, few publications on its usage were found. Moreover, it has not been translated into any other language. It is a self-administered questionnaire that is answered on designated computer tablets. It was selected from among other measurement tools owing to its good psychometric properties, its focus on similar target groups, namely inpatients, and because it also assesses the relational aspects of care among healthcare providers as a whole rather than a particular, individual provider. 

### 2.3. Translation and Cultural Adaptation of the RAC-Q

Before the RAC-Q underwent translation and cultural adaptation, permission to use the questionnaire was obtained from the corresponding author. The translation process followed the 10 steps proposed by established guidelines [13]. The steps were as follows below.

(1) *Preparation*

This initial phase involved the members of the research team members brainstorming about the translation and validation process, including three public health physicians and the main researcher. The translators, field experts, sampling population, and mode of data collection were meticulously discussed, considering the standard operating procedures and restrictions during the MCO due to COVID-19.

(2) *Forward Translation*

This step focused on translating the English version of the RAC-Q into the Malay language. Two independent translators were recruited to perform the task. The first translator is a professional translator with credible experience in translating medical-based materials and appointed by the Linguistic Department of Universiti Sains, Malaysia. The second translator is a medical doctor with a public health qualification and has vast experience in quality assurance programs in healthcare. Both translators are native Malay speakers and can converse in English fluently. Each translator carried out the translation independently, and they were asked to notify the team if they encountered any difficulties in translating any word or sentence from the original questionnaire.

(3) *Reconciliation*

This step aimed to produce a single forward translation by comparing and merging both forward translations of the translators. A committee consisting of experienced personnel working in hospital management, quality of care, nursing and clinical care, and language teaching was formed in order to review both forward translations. Both versions of the forward translations were compared with the original RAC-Q. The reconciliation process was completed by comparing each sentence in both translations. Words or phrases not relevant within the Malaysian context were replaced with alternative words or phrases. The content validity of the questionnaire was also determined at this stage. At the end of the meeting, one standard forward translation of the RAC-Q was produced and agreed upon by the review committee for its use in the next stage.

(4) *Back Translation*

Next, two independent translators translated the reconciled Malay version of the RAC-Q into English. This step aimed to compare the back-translated questionnaire with the original questionnaire and determine whether there were changes to the words or meanings. The first translator is a professional translator appointed by the Linguistic Department of Universiti Sains, Malaysia (different from the forward translator). The second translator was a postgraduate student enrolled in a public health course at Universiti Sains, Malaysia. Both are fluent in both languages and were blinded to the original questionnaire.

(5) *Back Translation Review*

At this stage, the same committee reviewed the back-translated questionnaire and compared it with the original English version. They examined the equivalence in terms of the concepts, items, and semantics of both versions. The conceptual equivalence assessment aimed to ascertain whether the theme of compassionate care in healthcare was present in both settings, whereas the item equivalence was examined to determine whether or not specific items were relevant and acceptable to both populations [15]. Semantic equivalence, on the other hand, was assessed to ensure that the original and translated questionnaires had similar meanings. Generally, the review committee was satisfied with the forward translation of the RAC-Q. Thus, the standard forward translation was agreed upon as the preliminary RAC-Q Malay version (RAC-QM) to be used in the next stage.

(6) *Harmonization*

An additional quality check was carried out to ensure that the back-translated RAC-QM was in harmony with the original English version. It was performed in order to detect any translation discrepancies between the two languages.

(7) *Cognitive Debriefing*

Cognitive debriefing was performed in order to test the translated version’s understandability, interpretation, and cultural relevance among a small group of relevant respondents. This enables one to detect any item that may be inappropriate and identify any other issues that may be confusing or misleading [16]. The cognitive debriefing was carried out by six respondents, namely, two males and four females in the age range from 30 to 45. Four of the respondents were inpatients who actually fulfilled the study criteria, and the other two respondents were nurses who worked in the wards. The respondents were briefed about the purpose of the cognitive debriefing. The preliminary RAC-QM was given to each respondent, and they were given time to answer all of the questions. They were then asked to give feedback on any confusing, unclear, or misleading sentences. They were also asked about their impression and understanding when they initially read particular phrases.

(8) *Review of Cognitive Debriefing Results and Finalization*

At this stage, feedback from the cognitive debriefing was presented and discussed with the review committee. The committee evaluated all of the comments, and necessary changes were made.

(9) *Proofreading*

Proofreading was performed as a final step to consolidate the RAC-QM and inspect it for grammatical or typographical errors. Lastly, the committee conducted a final check to ensure that any corrections had been made.

(10) *Final Report*

Lastly, a final report was created in order to document the whole process of the translation. This stage is essential for future translations and to ensure harmonization with the previously developed original-language version. 

### 2.4. Pretesting of RAC-QM

Pretesting was conducted at a teaching hospital and involved 30 adult inpatients that fit the study participants’ criteria. This step was carried out in order to further evaluate any shortcoming related to the questionnaire from the target population’s viewpoint. The RAC-QM was distributed to the participants through a QR code linked to a brief introductory video about the study.

### 2.5. Statistical Analysis

Data were analyzed using R software version 4.1.3 (R Core Team: Vienna, Austria, 2020). Owing to the non-normality of the data distribution, the robust maximum likelihood was preferred [17]. The construct validity was determined using CFA, and the internal reliability consistency was assessed using Cronbach’s alpha. Standardized factor loadings were measured, and items with factor loadings of >0.3 were considered acceptable [18]. The models’ goodness of fit was assessed using SRMR, RMSEA, CFI, and chi-squared tests following the values recommended by Schreiber et al. [19] and Brown [20]. The following cut-off values were taken into consideration to determine the goodness fit of the model: the comparative fit index (CFI) and Tucker–Lewis fit index (TLI) of >0.90, and the root mean square error of approximation (RMSEA) and standardized root mean square residual (SRMR) of <0.08 [17]. The revision of the model was considered on the basis of the factor loadings, standardized residuals (SRs), modification indices (MIs), and theoretical justification. Parameters with SR ≥ 2.58 and MI ≥ 3.84 were considered for the model. Factors were checked for multicollinearity if r > 0.85. The model was also selected on the basis of the Akaike Information Criterion (AIC) and Bayesian Information Criterion (BIC). Models with low values of the AIC and BIC were chosen [21]. A Cronbach’s alpha of ≥0.7 in the reliability assessment was considered acceptable [17].

### 2.6. Ethical Consideration

Ethical clearance for this research was obtained from the Human Research Ethics Committee of Universiti Sains, Malaysia (JEPeM Code: USM/JEPeM/21030208), and the National Medical Research Register (NMMR) Malaysia (NMRR-21-344-58027). The confidentiality of the data was strictly prioritized.

## 3. Results

Data from 138 inpatients were eligible for analysis. Sociodemographic characteristics of the patients were described quantitatively, and CFA was performed in order to validate the RAC-QM. 

### 3.1. Sociodemographic Characteristics of Inpatients

Initially, 150 respondents were recruited from six adult wards: the surgical ward, medical ward, orthopedic ward, oncology ward, obstetrics and gynecology ward, and a multidisciplinary executive ward. Of all the responses from 150 respondents, those from 12 respondents had to be removed owing to missing data.

With regard to the sociodemographic background of the respondents, there was an almost equal proportion of males and females. Half of them were aged below 40 years old, and the majority were Malays and Muslim. About one-quarter of the respondents did not have legal partners at the time of the study. More than 60 percent of the respondents completed secondary school, and almost 60 percent of the respondents were in the B40 income category, as classified on the basis of the Household Income and Basic Amenities survey of 2019, Department of Statistics, Malaysia [22]. In Malaysia, household income can be classified into three categories: B40, M40, and T20. B40 represents the bottom 40%, which, as of 2021, means a monthly household income of RM 4850 or less. M40 represents the middle 40%, which means a household income of RM 4851–10,970. T20 represents the top 20%, which can be further divided into T1 (household income ranging from RM 10,971 up to 15,040) and T2 (more than RM 15,041). Table 1 shows the details of the sociodemographic finding.

### 3.2. Medical and Admission Background

The patients’ medical background and details of their admission at the time were also gathered. The median admission period was four days, with an IQR of 8.25 days. Half of the patients had at least one chronic disease. The majority of the patients lived with other family members at home. However, only half of them were accompanied all the time while in the ward. More than 80 percent of them did not receive any visitors while in hospital. For almost 40 percent of the patients, this was the first time in their lives that they had ever been admitted to the ward. About half of the patients were dependent on others to move, practice self-care, or perform activities of daily living. About 60 percent of the patients experienced pain or discomfort. However, more than half reported no anxiety or depression issues. Table 2 summarizes all the findings.

### 3.3. Patients’ Responses to Questionnaire 

Table 3 shows the patients’ responses to the questionnaire. The questionnaire contained 12 items. Five of the items had three answer options, whereas the other seven questions had four answer options. All questions except for numbers four and twelve provided answer options in agreement form. For question numbers four and twelve, answer options were in frequency form. For the answers in agreement form, four points were given for “definitely true”, three points were given for “mostly true”, one point was given for “definitely false”, and two points were given for “unsure”. For the answers in frequency form, four points were given for “every time”, three points were given for “sometimes”, one point was given for “never”, and two points were given for “unsure”. 

Therefore, the maximum score a patient can provide is 48 points, and the minimum is 12 points. The total score that a patient provides is divided by 48 and multiplied by 100 to convert it to percent. The higher the score is, the more compassionate the healthcare provider is, as perceived by the patients. In general, the average points given by the patients for each item were between 3.42 and 3.91. The lowest points were given for question number one: “Did staff members introduce themselves before treating or caring for you?”. At the same time, patients could appreciate the staff members’ competency and gave them, on average, 3.91 points out of 4.0. 

### 3.4. Translation and Validation of RAC-Q

The translation and validation processes was performed according to the 10 proposed steps. In general, there was no significant difficulty faced during the process. The forward translation was carried out by two independent translators, who produced comparable and highly similar forward translations. An important point that was discussed was the appropriate Malay term for compassion. Both translators chose “penyayang” for “compassion”. Deeper consideration was given, as one of the central values of MOH’s corporate culture is “penyayang”, but it was translated into English as “caring”. After an in-depth discussion about the connotations of “budaya penyayang” in MOH’s corporate culture, the committee agreed that “penyayang”, in the corporate culture, is equivalent to compassion. “Penyayang” has also been used in the local healthcare system since the beginning of its service. Moreover, the concept of compassion has emerged relatively recently. No major modification was applied during the cognitive debriefing. The respondents gave feedback indicating that the questionnaire could be easily understood. The questions also sufficiently covered their thoughts about compassion. Some of the respondents suggested providing a short video to explain the survey before the respondents started to answer. Thus, a short introductory video was prepared and distributed during the pretest and actual survey.

### 3.5. Confirmatory Factor Analysis of the RAC-QM

**Figure 2:**
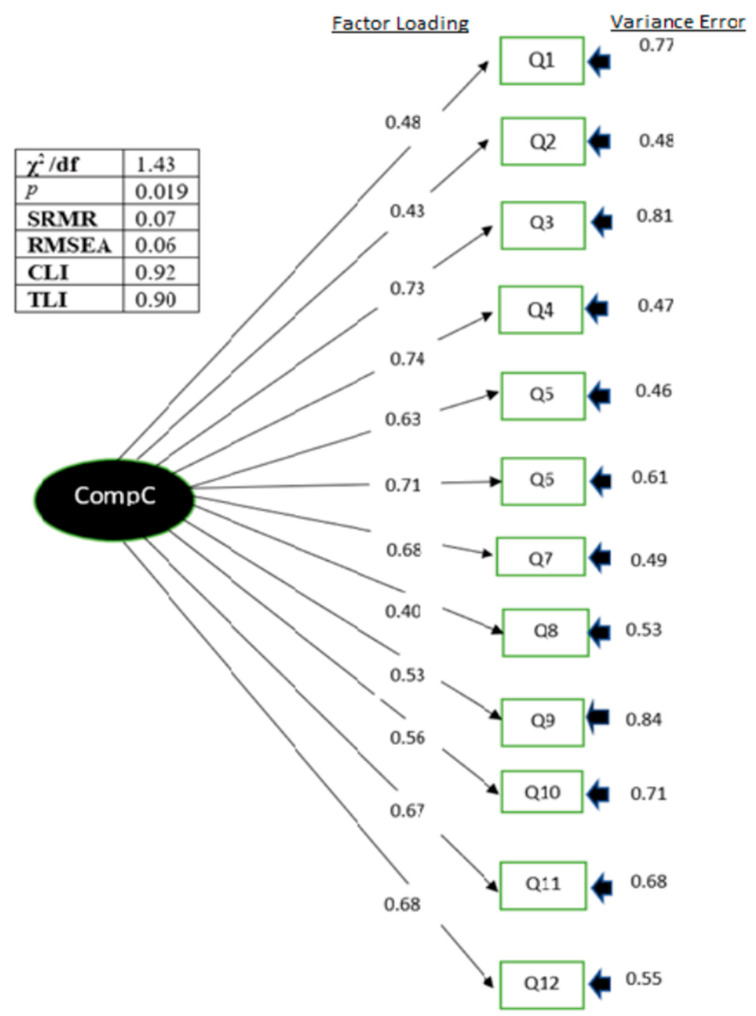
Path diagram of RAC-QM showing factor loadings and variance errors of each item.

The construct validity of the initial model (model 1) was determined using CFA. Model 1 contains items similar to the original version. In general, model 1 had a reasonable goodness of fit, with a chi-square statistic divided by the degree of freedom of 1.43, SRMR of 0.07, RMSEA of 0.06, CFI of 0.92, and TLI of 0.90. Model 1 also showed good reliability, with a Cronbach’s alpha of 0.85. The standardized factor loadings for all items were >0.40, except that for question number eight, which was 0.40, as shown in Table 4. Model 2 was created by excluding question number eight. Overall, model 2 did not have significantly better indices, and removing question number eight did not improve any other item’s factor loading, as shown in Table 5. Figure 2 shows the path diagram of the RAC-Q. There was no multicollinearity between items, and all the factor loadings were acceptable. 

## 4. Discussion

A healthcare organization is and has always been known as a place to relieve human suffering. However, assessments of medical care have traditionally been conducted based on technical and physiological reports of outcomes rather than from a patient’s perspective [21]. However, it is comforting to see that more healthcare systems all over the world have sought to achieve balance in the services that offer clinically effective and evidence-based care and that are also perceived by patients as acceptable and beneficial [21]. To properly assess a patient’s perception of the relational aspect of care that they experience, a valid measurement tool must be available, as suggested by a well-known report [11]. Reviews showed that most of the available patient-reported experience measures are still lacking in their psychometric properties [23]. Compassionate care is one of Malaysia’s MOH pillars. However, no tool has been psychometrically developed in order to assess whether this aspect of care is properly embedded among all healthcare workers. Therefore, in this study, we aimed to validate and translate the RAC-Q into the Malay language to enable its use in Malaysia. 

Although there are many other tools available that can be used to measure compassion, the RAC-Q was primarily chosen because it has very good psychometric properties, and the background of the original study population was similar to the targeted population of our study, that is, inpatients. Furthermore, the RAC-Q was developed on the basis of the relational aspect of care, based on evidence gathered from a wide selection of existing surveys, namely, the PEECH measure, NHS Adult Inpatient Survey, 2012 NHS Emergency Department Survey, CARE measure, Hospital Consumer Assessment of Healthcare Providers and Systems (HCAPS) questionnaire, and General Practice Patient Survey (GPPS), and also from a qualitative study [11]. It incorporates 22 elements of the relational aspect of care. Even though the original paper did not provide details about the specific themes that each question represented, the committee members tried to identify these during the cognitive debriefing and agreed that the questionnaire represented the 22 themes. 

Model 1 contained all 12 original items, with good indices (χ2/df = 1.43, SRMR = 0.07, RMSEA = 0.06, CFI = 0.92, and TLI = 0.90) and a good reliability, with a Cronbach’s alpha of 0.86. All factor loadings were also acceptable, with a value of >0.40, except for question number eight, the item that asks “have you had enough time to discuss your health or medical problem with a doctor or nurse?”. Therefore, Model 2 was built and tested by removing question number eight in order to decide which model has the better goodness of fit. Compared with model 1, model 2 has no significant difference in terms of the indices, although it leads to lower AIC (1006.14 vs. 1137.52) and BIC (1070.54 vs. 1207.77)). Therefore, model 1 was retained. According to Hair et al., a factor loading of >0.3 is still acceptable [17]. In addition, removing an important item from the questionnaire affects the intended functionality of the questionnaire. The reliability of the RAC-QM in assessing the compassionate care offered by healthcare providers was ascertained based on a good Cronbach’s alpha of ≥0.7. The overall consistency of the items in the questionnaire indicates that all the items measure the same constructs. The convergent validity of the questionnaire is also adequate, with a composite ratio of 0.857 and AVE of 0.344. Even though many studies suggested that the AVE should be more than 0.5, many lines of evidence also showed that a measurement model with a composite ratio of more than 0.6 is still adequate even if the AVE is less than 0.5 [24,25]. This means that similar results can be expected even if the testing process is repeated. 

As seen in the case of many other patient-reported experience measures, this survey also showed a ceiling effect, in which the majority of the responses skewed towards higher scores, that is, three and four. Even though this may indicate that the patients perceived all the healthcare workers they encountered as compassionate, research studies showed that their judgement could be influenced by many factors, such as social desirability bias, as a sign of appreciation, respect, deference, or generosity [26]. This small variation in the responses could pose a challenge when determining which areas require focus for improvement. That being said, it does not necessarily mean that the questionnaire could not be used to assess the problem that it was intended to address or that improvements could not be made. A skewness towards high scores was also observed in the original paper. Despite this, the authors were still able to make improvements and had a significant, positive outcome [11]. The responses could be analyzed item-by-item, and the questions that did not receive a full score could be treated as areas requiring attention and improvement. In this sense, we can treat the responses as dichotomous, that is, we can define a full score as “compassionate” and anything less as “not compassionate”. This has been practiced in the case of many patient-reported measures in view of the high prevalence of the ceiling effect [27,28]. In this study, we found that only question numbers seven and 10 had ceiling effects of more than 90 percent. There are several methods known to reduce the ceiling effect, even though they do not have strong evidence. These methods include removing neutral responses [29,30], making the responses more extreme [31], making statements regarding anonymity and the need for feedback in order to help future patients [32], changing the scale type, and applying the iterative Guttman-style scale [33,34]. These methods could be tested in future studies. 

The RAC-QM is a 12-item, self-administered questionnaire and relatively easy to understand. Even though it is short and straightforward, it is proven to measure compassionate care, as it was intended to do. This feature improves its clinical utility. As in the original study, this study also involved patients from multidisciplinary wards who had heterogeneous backgrounds. Thus, the generalizability and representativeness of the data can be viewed as a strength of this study. The ease of administration might also contribute to the clinical utility of the RAC-QM. Although the original version of the questionnaire originated from a western country, the RAC-QM still maintains good psychometric properties, which are attributable to the rigorous methods of translation and validation. 

This study should be considered in light of its limitations. Firstly, the study was carried out during the MCO due to COVID-19 in Malaysia. Most of the discussions were conducted virtually through calls, online discussions, and video conferences. Different methods of discussion or meeting may lead to different outcomes [35,36,37]. The study was also conducted in a university hospital and involved non-paying patients who were mostly unemployed, not highly educated, and had repeated hospitalizations, which may have altered their expectations of the free service they received. In addition, during the MCO, visitors were not allowed in the wards, and a companion was only allowed in special cases. In this sense, the patients depended entirely on the staff members for their treatment and care. Appreciation, respect, and generosity may have affected their responses as well. 

## 5. Conclusions

In conclusion, the RAC-QM was proven to have good psychometric properties and can be used in Malaysia in order to measure the compassion level of healthcare providers from the patient’s perspective. With the availability of this tool for measuring the level of compassionate care, MOH can start to evaluate this crucial aspect of quality care and carry out the necessary improvements. 

## Figures and Tables

**Table 1:** Sociodemographic characteristics of the patients (*n* = 138).

Characteristics	*n* (%)
Gender	
Male	71 (51.4)
Female	67 (48.6)
Age	
18–39	69 (50.0)
40–59	36 (26.1)
60 and above	33 (23.9)
Race	
Malay	130 (94.2)
Chinese	6 (4.3)
Siamese	1 (0.7)
Other	1 (0.7)
Religion	
Islam	131 (94.9)
Christianity	1 (0.7)
Buddhism	6 (4.3)
Marital status	
Married	103 (74.6)
Single	24 (17.4)
Separated	11 (8.0)
Education level	
No formal education	3 (2.2)
Primary	13 (9.4)
Secondary	69 (50.0)
Tertiary	49 (35.5)
Postgraduate	4 (2.9)
Occupational status	
Government worker	31 (22.5)
Private sector	15 (0.9)
Self-employed	26 (18.8)
Student	13 (9.4)
Pensioner	14 (10.1)
Not employed	39 (28.3)
Household income	
Less than RM 4850	79 (57.2)
4850–10,959	25 (18.1)
10,960–15,039	4 (2.9)
>15,040	1 (0.7)
No income	29 (21.0)

**Table 2:** Medical and admission background (*n* = 138).

Characteristic	*n* (%)	Mean (SD)	Median (IQR)
Day(s) of admission			4 (8.25)
Distance from home (km)			19 (24)
Self-rated health status score		74.6 (15.5)	
Chronic illness None One disease More than one	62 (44.9) 38 (27.5) 38 (27.5)		
Self-rated health Healthy Not satisfactory	117 (84.8) 21 (15.2)		
Not alone at home Lives alone	132 (95.7) 6 (4.3)		
Companion in ward All the time Occasionally None	75 (54.3) 18 (13.0) 45 (32.6)		
Visitors while in ward Everyday Occasionally None	5 (3.6) 17 (12.3) 116 (84.1)		
First hospital admission First hospitalization Repeated hospitalization	54 (39.1) 84 (60.9)		
Reason for admission Emergency Elective	83 (60.1) 55 (39.8)		
Ability to move No problem Some problems Bedbound	73 (52.9) 52 (37.7) 13 (9.4)		
Ability to practice self-care No problem Some problems Unable	80 (58.0) 43 (31.2) 15 (10.9)		
Activities of daily living No problem Some problems Unable	56 (40.6) 56 (40.6) 26 (18.8)		
Pain or discomfort None Some A lot	53 (38.4) 77 (55.8) 8 (5.8)		
Anxiety or depression None Some Severe	93 (67.4) 39 (28.3) 6 (4.3)		

**Table 3:** Responses to the questionnaire (*n* = 138).

Statements	Total Score Mean (SD)	Responses *n* (%)				Min, Max
		(1)	(2)	(3)	(4)	
1. Did staff members introduce themselves before treating or caring for you?	3.42 (0.80)	9 (6.5)	NA	53 (38.4)	76 (55.1)	1, 4
2. Have staff members taken the opportunity to learn about you as a person?	3.49 (0.69)	5 (3.6)	0 (0)	56 (40.6)	77 (55.8)	1, 4
3. Have staff members made you feel at ease by being friendly and warm in conversations?	3.81 (0.39)	0 (0)	NA	26 (18.8)	112 (81.2)	3, 4
4. Have staff members showed you care and compassion?	3.74 (0.49)	1 (0.7)	0 (0)	33 (23.9)	104 (75.4)	1, 4
5. Have staff members listened to what you have to say?	3.90 (0.30)	0 (0)	NA	14 (10.1)	124 (89.9)	3, 4
6. During your time in the hospital, have you had enough contact with staff members?	3.83 (0.38)	0 (0)	NA	24 (17.4)	114 (82.6)	3, 4
7. Do staff members appear confident and able to perform their tasks when caring for you?	3.91 (0.28)	0 (0)	0 (0)	12 (8.7)	126 (91.3)	3, 4
8. Have you had enough time to discuss your health or medical problem with a doctor or nurse?	3.80 (0.46)	1 (0.7)	0 (0)	25 (18.1)	112 (81.2)	1, 4
9. Have you been involved as much as you want to be in decisions about your care and treatment?	3.81 (0.45)	1 (0.7)	0 (0)	23 (16.7)	114 (82.6)	1, 4
10. During your time in the hospital, have staff members made you feel safe?	3.89 (0.38)	1 (0.7)	0 (0)	12 (8.7)	125 (90.6)	1, 4
11. Have you received as much support as you have needed from staff members?	3.80 (0.46)	1 (0.7)	0 (0)	25 (18.1)	112 (81.2)	1, 4
12. Overall, do you feel that you have been treated with respect and dignity while in the hospital?	3.90 (0.30)	0 (0)	NA	14 (10.1)	124 (89.9)	3, 4
Total	3.78 (0.450	19 (1.15)	0 (0)	317 (19.1)	1329 (80.3)	

(1) Definitely false or never; (2) unsure; (3) mostly true or sometimes; (4) definitely true or every time.

**Table 4:** Factor loading and internal consistency reliability of RAC-QM.

Item	Factor Loading	Cronbach’s Alpha	Composite Ratio
Q1	0.48	0.857	0.857
Q2	0.43		
Q3	0.73		
Q4	0.74		
Q5	0.63		
Q6	0.71		
Q7	0.68		
Q8	0.40		
Q9	0.53		
Q10	0.56		
Q11	0.67		
Q12	0.68		

**Table 5:** Confirmatory factor analysis of RAC-QM.

Model	χ^2^ (df)	χ^2^/df	*p*	SRMR	RMSEA	90% CI	CFI	TLI	AIC	BIC
Model 1	77.7 (54)	1.43	0.019	0.07	0.06	0.03, 0.08	0.92	0.90	1137.52	1207.77
Model 2	59.4 (44)	1.35	0.059	0.07	0.05	0.02, 0.07	0.94	0.93	1006.14	1070.54

CFI: comparative fit index; TLI: Tucker–Lewis fit index; AIC: Akaike information criterion; BIC: Bayesian information criterion; SRMR: standardized root mean square residual; RMSEA: root mean square error of approximation.

## Data Availability

The data are not publicly available due to privacy and confidentiality. However, restrictions apply to the availability of the hospital data, which are available from the authors with the organization’s permission.
